# Lung protective vs. standard ventilation during laparoscopic surgery in obese patients. preliminary results of a randomized, controlled trial

**DOI:** 10.1186/2197-425X-3-S1-A683

**Published:** 2015-10-01

**Authors:** DL Grieco, A Russo, MS Vallecoccia, L Polidori, B Costantini, V Simili, F Varone, G Scambia, E Marana, M Antonelli

**Affiliations:** Anesthesiology and Intensive Care Medicine, Catholic University of Rome, Rome, Italy; Gynaecology and Obstetrics, Catholic University of Rome, Rome, Italy; Pulmonary Medicine, Catholic University of Rome, Rome, Italy

## Introduction

Two different papers published recently suggested the use of a comprehensive strategy providing low tidal volumes, peep and recruiting maneuvers in patients undergoing open abdominal surgery (1,2). It is unknown whether such ventilatory approach may be feasible in patients undergoing laparoscopy, as pneumoperitoneum and Trendelenburg position may alter lung volumes and chest-wall elastance.

## Objectives

We designed an open-label randomized, controlled trial to assess the effect of a lung-protective ventilation strategy in obese patients undergoing laparoscopic surgery.

## Methods

ASA status I-II morbidly obese patients(BMI>35) undergoing gynecological laparoscopic surgery were randomly assigned to intraoperative volume-controlled protective(TV6 ml/kgIBW, peep10, recruiting maneuvers)(PV) or standard(TV10 ml/kgIBW, peep 5) ventilation (SV). An esophageal catheter was placed to estimate pleural pressure and compute transpulmonary pressure. Results are expressed as median[interquartile range].

## Results

Twelve patients were enrolled (age 62[57-67], BMI 44[39-48], IBW 52[46-58], length of surgery 185[165-209]minutes).

Intraoperative PaO2/FiO2 was not different between groups(p = 0.33), whereas mean PaCO2 and respiratory rate were lower and mean pH was higher in SV patients (35[34-36]vs.41[37-42], p = 0.05; 14 mmHg[13-17]vs.25 mmHg[21-27], p = 0.04; 7.42[7.40-7.43]vs.7.37[7.36-7.39], p = 0.01). During pneuomoperitoneum, patient in PV group showed a lower transpulmonary driving pressure (8.5[7-10]cmH2O vs. 14[12.5-20]cmH2O, p = 0.007) and a trend to a higher lung compliance (40[36-57]ml/cmH2O vs. 29[19-40]ml/cmH2O, p = 0.08). During pneumoperitoneum, in none of the two groups positive end expiratory pressure was able to generate a positive transpulmonary end-expiratory pressure (PV -1[-5-0]cmH2O and SV -6[-10 - -4]cmH2O). PaO2/FiO2, respiratory rate, PaCO2 and pH 1 hour and one day after extubation were not different between groups. Comparison of pulmonary function tests at day 2 showed similar FEV1 and FEV1/FVC ratio, while a higher percentage of predicted forced vital capacity was detected in patients of SV group(100%[83-110]vs.78%[71-88];p = 0.04).

## Conclusions

Preliminary results of the present randomized controlled trial indicate that a comprehensive lung-protective strategy providing low tidal volumes, higher peep and recruiting maneuvers during laparoscopic surgery in obese patients, despite optimizing intraoperative respiratory mechanics, may not yield a relevant benefit on postoperative oxygenation and respiratory function.
